# GWAS for Meat and Carcass Traits Using Imputed Sequence Level Genotypes in Pooled F2-Designs in Pigs

**DOI:** 10.1534/g3.119.400452

**Published:** 2019-07-11

**Authors:** Clemens Falker-Gieske, Iulia Blaj, Siegfried Preuß, Jörn Bennewitz, Georg Thaller, Jens Tetens

**Affiliations:** * Department of Animal Sciences, Georg-August-University, 37077 Göttingen, Germany; † Institute of Animal Breeding and Husbandry, Kiel University, 24118 Kiel, Germany; ‡ Institute of Animal Husbandry and Breeding, University of Hohenheim, 70599 Stuttgart, Germany, and; § Center for Integrated Breeding Research, Georg-August-University, 37077 Göttingen, Germany

**Keywords:** Genome wide association study, Whole genome sequencing, Imputation, Meat, carcass, and production traits, Variant calling

## Abstract

In order to gain insight into the genetic architecture of economically important traits in pigs and to derive suitable genetic markers to improve these traits in breeding programs, many studies have been conducted to map quantitative trait loci. Shortcomings of these studies were low mapping resolution, large confidence intervals for quantitative trait loci-positions and large linkage disequilibrium blocks. Here, we overcome these shortcomings by pooling four large F2 designs to produce smaller linkage disequilibrium blocks and by resequencing the founder generation at high coverage and the F1 generation at low coverage for subsequent imputation of the F2 generation to whole genome sequencing marker density. This lead to the discovery of more than 32 million variants, 8 million of which have not been previously reported. The pooling of the four F2 designs enabled us to perform a joint genome-wide association study, which lead to the identification of numerous significantly associated variant clusters on chromosomes 1, 2, 4, 7, 17 and 18 for the growth and carcass traits average daily gain, back fat thickness, meat fat ratio, and carcass length. We could not only confirm previously reported, but also discovered new quantitative trait loci. As a result, several new candidate genes are discussed, among them *BMP2* (bone morphogenetic protein 2), which we recently discovered in a related study. Variant effect prediction revealed that 15 high impact variants for the traits back fat thickness, meat fat ratio and carcass length were among the statistically significantly associated variants.

Mapping experiments in livestock generally serve two purposes: The first is to understand the genetic architecture of quantitative traits, and to derive and prove new hypotheses of trait expression. The second is the identification of genetic markers that may be useful for livestock breeding. There have been many quantitative trait loci (QTL) mapping experiments carried out over the last decades (see review article by (Rothschild *et al.* 2007)), mainly in experimental F2 crosses established from two outbred founder pig breeds. In early studies, genotyping was mainly achieved using microsatellite markers and mapping was achieved through linkage analysis (see overview in (Knott 2005)). These designs were set up to enable QTL detection with high power, but they suffered from a low mapping resolution and large confidence intervals for QTL-positions. This was partly due to the limited number of meiosis cycles exploited in these designs in conjunction with typically small numbers of 300 to 500 F2 individuals. Furthermore, this approach assumes the divergent fixation of the QTL alleles in the founder breeds, and highly different gene frequencies and variation within these breeds were not considered (Nagamine *et al.* 2003). The breed Piétrain, for instance, has been selected for growth and meat yield for many generations and still exhibits a large genetic variation for these traits (Wellmann *et al.* 2013). More recent QTL-mapping experiments utilized genome-wide association studies (GWAS), which in contrast to linkage analyses, exploit historical meiosis and rely on linkage disequilibrium (LD) requiring high marker densities. The precision of GWAS is then a function of LD block lengths and the number of individuals analyzed, which in turn limits the usefulness of its application in F2 designs (Hayes and Goddard 2001). However, enormous efforts have been made in the establishment of these mapping populations, usually including extensive phenotyping far beyond what would be available in field populations. It would thus be desirable to revisit these resources using current genotyping and sequencing technologies, which would require an increase in the number of individuals and a decrease in the LD block lengths. In a recent simulation study, it was shown to be possible by pooling F2 designs, particularly when founder breeds are closely related and QTL are segregating in one founder breed (Schmid *et al.* 2018). This approach has already been successfully applied based on medium density SNP chip data (Blaj *et al.* 2018; Stratz *et al.* 2018).

With the aim to overcome the aforementioned limits in mapping resolution and to fully exploit the potential of the resource populations, we pooled four well-characterized F2 designs (Table 1), three of them having the founder breed Piètrain in common. Twenty four founder animals were genotyped by high coverage whole genome sequencing (WGS) and 91 of the F1 animals were sequenced at a low coverage for subsequent imputation to a high coverage WGS level. A total of 2,657 F2 animals that were genotyped with the 62K Illumina PorcineSNP60 BeadChip (Ramos *et al.* 2009) were imputed to WGS levels with pedigree information and analyzed in a joint GWAS (see workflow in Figure 1). As a proof of concept four relevant production traits were analyzed: Average daily gain (ADG), back fat thickness (BFT), meat to fat ratio (MFR), and carcass length (CRCL).

**Table 1:** Per cross information of the sequenced individuals (F0 and F1) and SNP array genotyped individuals (F2). F0 and F1 animals served as the reference panel for the imputation of the F2 generation to sequence level for subsequent genome wide association analyses

Cross/Generation	F0*	F1	F2
Piétrain x (Large White x Landrace)/Large White	13	55	1750
Meishan x Piétrain	8	19	304
Wild Boar x Piétrain	6	17	291
Wild Boar x Meishan	1	0	312
**Total**	24*	**91**	**2657**

* Four founders are common among crosses.

**Figure 1:**
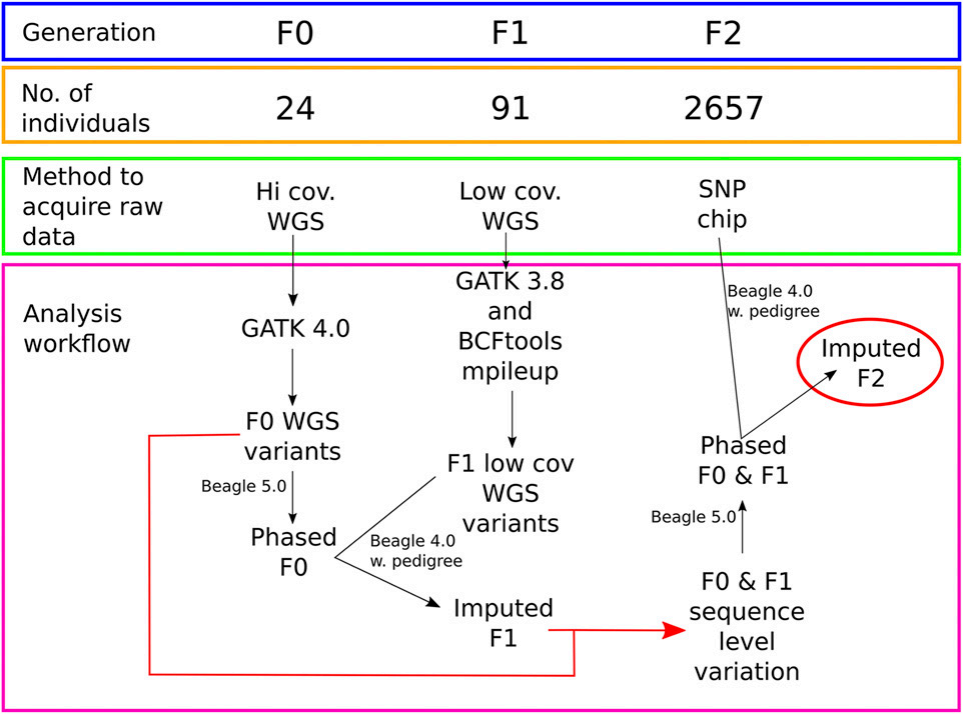
Genotyping workflow. 24 Founder animals were sequenced with high coverage, variants were called with GATK 4.0 and phased with Beagle 5.0. 91 F1 animals were sequenced with low coverage and variants were called with GATK 3.8 and BCFtools mpileup. The F1 dataset was imputed using Beagle 4.0 and pedigree information with phased Founders as a reference-panel for haplotype structure. The imputed F1 was then merged with the F0 variant call data set and phased with Beagle 5.0. Finally the 2657 chip genotyped F2 individuals were imputed to WGS levels with Beagle 4.0 and pedigree information with the merged and phased Founder/F1-imputed dataset as the reference-panel.

## Material and Methods

### Description of resource populations and phenotypes

Four well characterized experimental populations were pooled for this study. Detailed descriptions of the resource populations were done by Borchers *et al.* (Borchers *et al.* 2000) and Rückert *et al.* (Rückert and Bennewitz 2010), hence they will only be described briefly. The largest population was obtained from five purebred Piétrain boars and one Large White and six crossbred sows Landrace x Large White. The other three populations stemmed from a Meishan boar or Wild boar crossed with either Piétrain or Meishan females. The Wild boar and three Piétrain females were common founders in three of the crosses. The F2 generation was the result of repeatedly crossing F1 boars with F1 sows in order to obtain large full-sib families. From the crosses, a total number of 2772 animals were chosen (24 F0-generation pigs, 91 F1-generation pigs, 2657 F2-generation pigs) and blood samples were used to extract genomic DNA for genotyping purposes (Table 1). The F0 and F1 animals selected for sequencing were generally chosen according to the number of F2 individuals, *i.e.*, we prioritized individuals from which large families were derived. Four phenotypic traits were considered: ADG, BFT, MFR, and CRCL. The phenotypes were pre-corrected for systematic effects (*e.g.*, stable, slaughter month) and for the effect of *RYR1* gene (Fujii *et al.* 1991) using a general linear model. Trait definition, descriptive statistics and information about the pre-adjustment and the fixed effects used per cross can be found in (Blaj *et al.* 2018).

### Sequencing

A total number of twenty four founder animals were sequenced with an average 19x coverage at the sequencing facility University Hohenheim. Out of 17 F1 families, 91 animals were sequenced with an average 0.9x coverage. All paired-end sequencing (read length 2 × 100 bp) was done on an Illumina HiScan SQ using TruSeq SBS v3 Kits. For the library construction, the DNA samples were fragmented on a Covaris S220 ultrasonicator. Parameters were adjusted to yield 350 bp inserts. Fragment length was measured with High Sensitivity DNA Chips on an Agilent Bioanalyzer. Sequencing adapters and indexes were ligated using Illumina’s TruSeq DNA PCR-Free Library Prep Kits. Quantification of libraries was done by qPCR using KAPA Library Quant Kits. Flow cells were prepared using an Illumina cBot and TruSeq PE v3 Cluster kits. Raw sequencing data were demultiplexed and converted into FASTQ files using Illumina’s CASAVA software.

### Mapping and variant detection

Mapping and variant calling of the F0 generation was performed according to the GATK best practice pipeline using GATK v. 4.0 (McKenna *et al.* 2010) and genome assembly *Sus scrofa* 11.1 (GCA_000003025.6 provided by Swine Genome Sequencing Consortium on NCBI). Base quality score recalibration was performed with dbSNP build 150 as the knownSites dataset. Truth datasets used for Variant Quality Score Recalibration (VQSR) were as follows. SNPs: Illumina Infinium PorcineSNP60 v2 BeadChip and Affymetrix Axiom PorcineHD. INDELs: High confidence fraction (filter settings: QD 15.0, FS 200.0, ReadPosRankSum 20.0) of the PigVar database. Training dataset for SNP VQSR was also a high confidence fraction of the PigVar database (filter settings: QD 21.5, FS 60.0, MQ 40.0, MQRankSum 12.5, ReadPosRankSum 8.0, SOR 3.0) (Zhou *et al.* 2017). A truth sensitivity of 99.0 was chosen for SNPs and INDELs. The known dataset for SNP and INDEL VQSR was dbSNP build 150. Since SNPs were filtered with two truth datasets a Ti/Tv free recalibration according to the GATK best practice guidelines was applied to the data. Low coverage sequencing reads of F1 animals were processed according to the GATK best practice guidelines with the following deviations. SNP Calling was performed using GATK HaplotypeCaller v. 3.8 in joint mode with the settings minPruning 1 and minDanglingBranchLength 1 as well as BCFtools mpileup v 1.9 (Li *et al.* 2009), respectively. INDELs in the F1 variant call dataset were neglected due to low sequencing depth. An intersection variant call set between HaplotypeCaller, mpileup and the founder SNPs was created and stringently filtered with the following settings: QD 30.0, FS 60.0, MQ 40.0, QUAL 300.0.

### Haplotype construction and imputation

To make use of the most recent phasing algorithms Beagle 5.0 was used for all phasing operations (Browning *et al.* 2018). Beagle 4.0 was applied for genotype imputation since it is the latest version that supports the usage of pedigree information (Browning and Browning 2007). Haplotype phasing of the F0 generation variant call set was done using Beagle 5.0 and subsequent imputation with pedigree information of the F1 low coverage SNPs was achieved with Beagle 4.0. F0 and imputed F1 variants were merged with GATK CombineVariants and phased with Beagle 5.0. F2 generation 60k SNP chip data were imputed with Beagle 4.0 and pedigree information with merged and phased F0 and F1 WGS level variants as the reference panel. Local drops in imputation accuracy were determined by the construction of 24 F0 reference-panels with one animal left out. Genotype data acquired with the 60k SNP chip from each F0 individual was imputed with a reference-panel where the respective individual was missing utilizing Beagle 4.0. The 24 individual datasets were merged and together with the F0 reference dataset converted to additive coding with Plink 1.9 (Chang *et al.* 2015). Correlation (coefficient of determination, R^2^) for each variant on QTL harboring chromosomes was calculated with an in house R script.

### Genome wide association studies and cluster assignment

Single-trait association analyses were performed with GCTA v. 1.92.4 beta 3 on the F2 population only (Yang *et al.* 2011). In order to perform a “leave one chromosome out” (LOCO) analysis, multiple genomic relationship matrices (GRMs) were created from the F2 60k SNP chip data by excluding each chromosome once with a minor allele frequency (MAF) cutoff of 1%. Mixed linear model association analyses (MLMAs) were performed with imputed F2 variants for each chromosome separately using the GRM where the respective chromosome was left out and a MAF cutoff of 1%. To account for the pooled population structure, covariates representing the different crosses (4 classes) were included in the MLMA. For further downstream analysis, significance threshold was established by applying Bonferroni correction (*i.e.*, 0.05/number of independent tests). Manhattan plots were created with R, where variants with p-values > 0.001 were excluded due to software limitations. Clusters incorporating potential genomic regions of interest were defined using the Manhattan Harvester (MH) tool (Haller *et al.* 2019). MH provides quality assignment for each peak via a general quality score (GQS) which can be used as the main parameter for peak assessment. The GQS is generated based on a trained mixed-effects proportional odds model using 16 various parameters (*e.g.*, maximal slope, height to width ratio) and human peak identification data. For this study, the variants with a p-value below 1.0x10^−7^ (option *-inlimit*) were included and further the clusters with a GQS > 3.5 (1 is min and 5 is max) were taken into account. Conditional association analyses were performed by including single highly associated variants as fixed effects in a LOCO analysis.

### Variant effect prediction and gene enrichment analysis

To predict variant effects the Ensembl Variant Effect Predictor (VEP) release 94 was utilized, which is part of the Ensembl advanced programming interface (API) (McLaren *et al.* 2016). The vep command using the clusters’ statistically significant variants was executed with the following settings: *–merged –force_overwrite –variant_class –symbol –nearest gene*. To provide further functional interpretation, the Database for Annotation, Visualization and Integrated Discovery (DAVID) (Huang da *et al.* 2009) was used for a systematic and integrative analysis. The gene list from the VEP output was the input for DAVID (Huang *et al.* 2007) and *Sus scrofa* genes were considered as the background. Gene Ontology (GO) terms (*i.e.*, cellular component, molecular function, and biological process) from the functional annotation chart report which were significantly overrepresented with an EASE Score (*i.e.*, a modified Fisher Exact P-Value) below 0.05 and with a gene count higher or equal to 5 were retained.

### Statement on data and reagent availability

Sequencing data, which were used to conduct this study will be made publicly available upon publication of the article in the NCBI Sequence Read Archive (SRA). Supplementary Tables have been uploaded to GSA. Supplementary Table 1 contains the coefficients of determination (R^2^) for each variant on QTL harboring chromosomes where calculation was possible. Supplementary Table 2 containts the complete list of clusters identified in the GWAS with additional supporting information for cluster assignment. GRMs from F2 60k genotypes (File_S1.zip) were created by a “leave one chromsome out” approach using the program “Genome-wide Complex Trait Analysis (GCTA) version 1.91.4 beta3”. GWAS was conducted with imputed sequence level F2 genotypes (Supplementary File_S2.zip) for each chromosome using the GRM where the respective chromosome was left out(GRM command: gcta64–bfile SG_F2_chip_wo_chrNO–autosome–maf 0.01–make-grm–out SG_F2_chip_wo_chrNO–thread-num 10–autosome-num 18 ; GWAS command: gcta64–mlma–covar Kiel_Hoh_cross.covar–bfile F2_beagle4.0_ped_ChrNO–grm SG_F2_chip_wo_chrNO–pheno TRAIT.pheno–out TRAIT_chrNO–maf 0.01–thread-num 10; replace NO with the respective chromosome number and TRAIT with the respective trait to be analyzed) Phenotype files are located in Supplementary File_S2.zip for the traits ADG, BFT, MFR, and CRCL were used in the GWAS. Crosses were used as covariates in the GWAS and provided as a gcta compatible covar file in Supplemental File_S4.zip. SNP locations can be found in the bim files of the genotype data (Supplementary File_S1.zip and Supplementary File_S2.zip). Population structure information is provided in form of a Beagle 4.0 compatible pedigree file (Supplementary File_S3.zip). Raw sequencing data are accessible via the NCBI Sequence Read Archive (SRA) under BioProject ID PRJNA553106. File_S5 contains the 60k chip genotype data in variant call file (VCF) format. Genomic positions have been lifted to genome assembly *Sus scrofa* 11.1 (GCA_000003025.6) and annotated with dbSNP build 150. gcta compatible covar files for the conditional association analyses with top variants are provided in File_S6. Supplemental material available at FigShare: https://doi.org/10.25387/g3.8287847.

## Results

### Whole genome sequencing and variant calling

An average of 592,788,350 (SD = 38,623,216, MIN = 525,921,083, MAX = 649,442,924) sequencing reads per sample were aligned to the reference genome in the F0 generation with an average mapping efficiency of 99.37%. In the F1 generation an average of 26,562,876 (SD = 6,619,568, MIN = 15,915,385, MAX = 59,300,856) reads were mapped to the reference assembly with an average mapping efficiency of 99.33%.

With respect to the number of SNPs detected in the founder population, 22,671,759 were previously reported and 3,950,955 were novel. Furthermore, 1,482,139 of the INDELs were previously reported and 4,335,345 were novel. Per chromosome, average distances among the variants are summarized in Table 2. The Ti/Tv of SNPs in the founder population was 2.39, whereas known SNPs had a Ti/Tv of 2.44 and novel SNPs had a Ti/Tv of 2.08. Due to the low sequencing coverage (average = 0.96 x, min = 0.58 x, max = 2.14 x) only autosomal SNPs were called in the F1 population. The raw output of Haplotypecaller consisted of 20,055,697 known and 3,529,441 novel SNPs and the raw output of mpileup contained 19,932,201 known and 3,291,758 novel SNPs. The intersection of the two datasets resulted in 19,264,662 known and 2,951,058 novel SNPs whereas removing all SNPs that were not present in the founder variant calling dataset lead to a final number of 19,224,132 known and 2,911,780 novel raw SNPs. After the application of a stringent filtering approach (see Material and Methods) 5,753,444 known and 741,155 novel SNPs remained in the variant calling dataset of the F1 population.

**Table 2:** Average distance between variants discovered in the founder population. A number of 24 F0 animals were sequenced at high coverage and the average distances between variants (SNPs and INDELs) were calculated per chromosome

Chromosome	Avg. distance (bp)	SD
1	105,78729	196,8571
2	84,83889	201,4813
3	79,13779	183,627
4	80,16339	174,2767
5	73,37176	177,8913
6	85,34639	214,5176
7	79,12655	175,2067
8	78,90639	164,8448
9	79,61324	166,9157
10	56,5826	141,8648
11	67,6446	139,1412
12	65,41473	190,7484
13	102,70483	209,4908
14	83,58366	158,1296
15	93,02928	187,2321
16	71,18928	155,2467
17	65,91122	163,1281
18	76,95204	149,7542
		
**Mean**	**82,21004**	**180,6988**

### Identification of local drops in imputation accuracy

To detect local inaccuracies in the imputed data, we imputed chip data from each founder with the remaining 23 founders as a reference panel. The data does not provide information about the imputation accuracy of the experiment since pedigree information could not be used. The coefficient of determination for each variant located on a chromosome harboring relevant QTL was determined where feasible. The average coefficients of determination for each chromosome analyzed are summarized in Table 3 (complete analysis results in Supplementary Table 1).

**Table 3:** Identification of local imputation inaccuracies. Chip data from each of the 24 founders was imputed using the remaining 23 founder animals as the reference panel. Coefficients of determination (R^2^) were calculated for each variant in order to calculate average R^2^ for SSC1, SSC2, SSC4, SSC7, SSC17, and SSC18

Chromosome	Average R^2^	SD
1	0.28	0.32
2	0.22	0.29
4	0.25	0.30
7	0.25	0.31
17	0.18	0.25
18	0.29	0.32

### GWAS results and clusters

**Figure 2:**
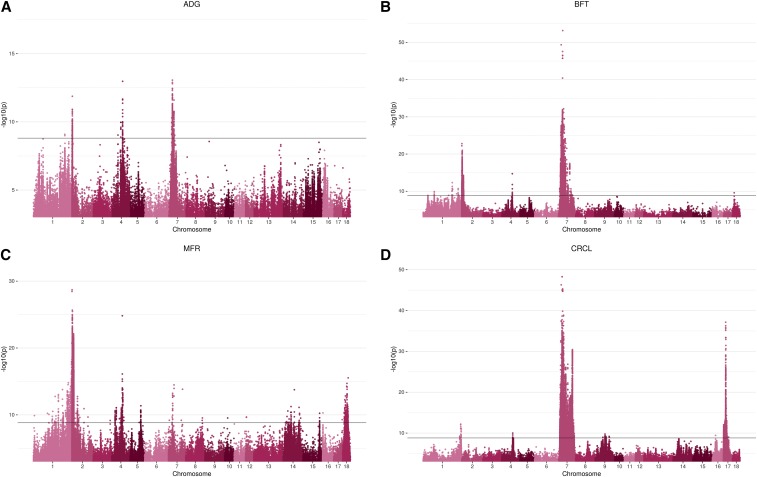
Manhattan plots of the −log_10_ p-values for association of variants with the traits (A) average daily gain (ADG), (B) back fat thickness (BFT), (C) meat to fat ratio (MFR), and (D) carcass length (CRCL). P-values > 0.001 were excluded from the plots.

**Figure 3:**
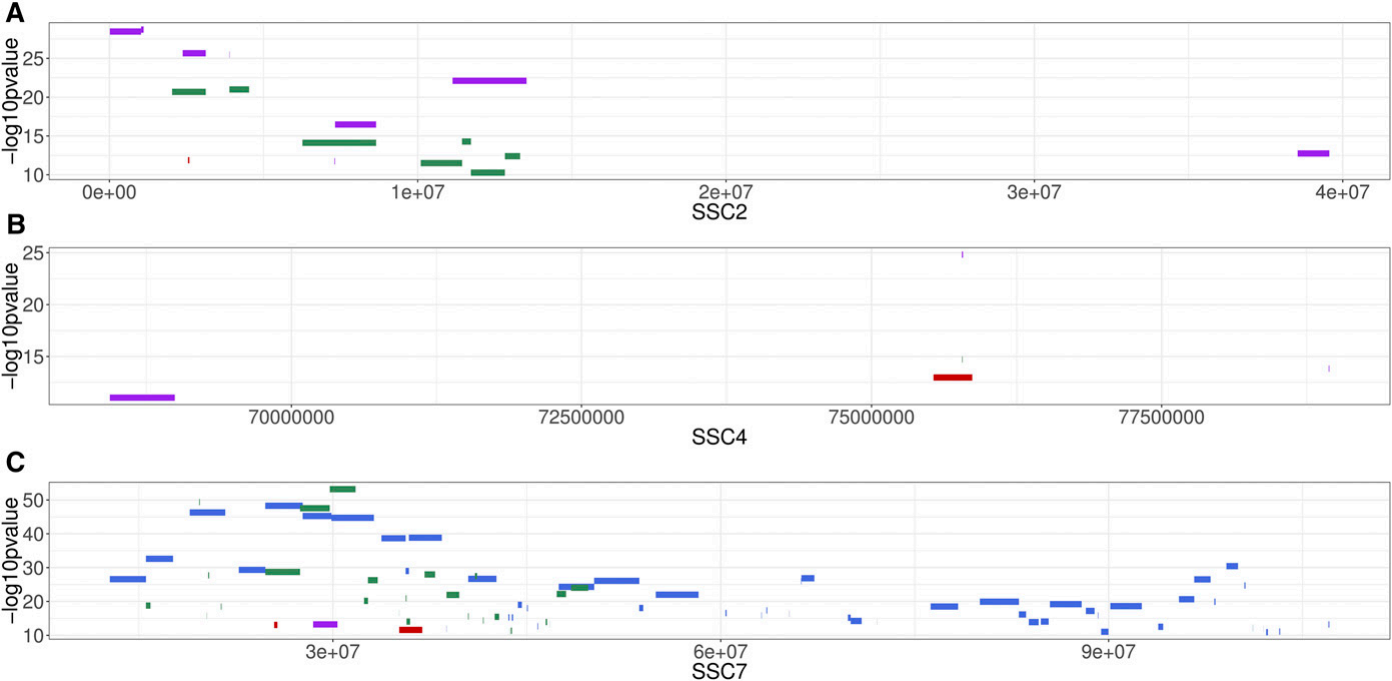
Cluster overlap for (A) SSC2, (B) SSC4 and (C) SSC7 for all traits (average daily gain (ADG) – red, back fat thickness (BFT) – green, meat to fat ratio (MFR) – purple, and carcass length (CRCL) - blue). The heights of the clusters are according to the top variant (– log_10_ p-value) within each given cluster.

**Figure 4:**
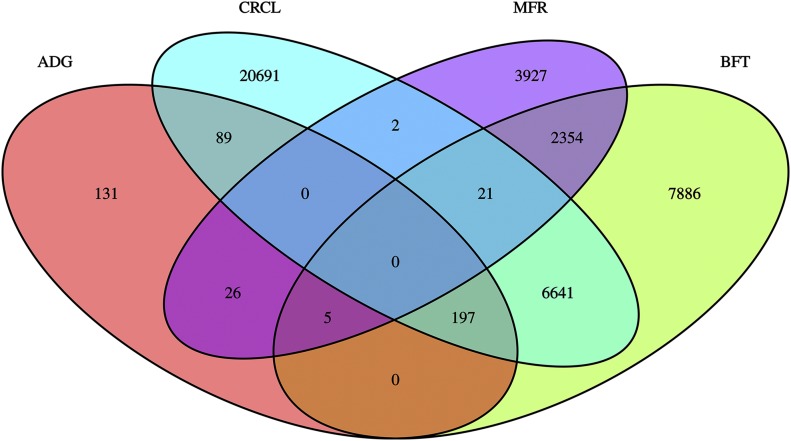
Variants concordance and discordance between the traits average daily gain (ADG), back fat thickness (BFT), meat to fat ratio (MFR), and carcass length (CRCL). The Venn diagram contains statistically significant variants. Intersections between traits include the number of common variants. Numbers of variants that were exclusively found in the single traits are outside of intersections.

From the genome-wide association study conducted in the pooled F2 population, the following number of variants exceeded the genome-wide significance threshold: 448, 17,105, 6635, and 27,641 for ADG, BFT, MFR, and CRCL, respectively. Manhattan plots of the GWAS for the four phenotypic traits are shown in Figure 2. A total of 120 clusters were designated by the MH tool (*i.e.*, 4 for ADG, 33 for BFT, 22 for MFR and 61 for CRCL) and they were located on the following *Sus Scrofa* chromosomes (SSC): 1, 2, 4, 5, 7, 17, and 18. The complete cluster list with additional supporting information for cluster assignment can be found in Supplementary Table 2. From each of the defined clusters, the top 5 variants were retained. The genes incorporating or lying nearby these highly significant associations are presented in Table 4. The clusters associated with the traits overlapped on several chromosomes, specifically on SSC2, SSC4, and SSC7. The location and the extent of the overlapping clusters is depicted in Figure 3. Particular chromosomes had exclusive clusters assigned, *e.g.*, SSC17 for CRCL and SSC18 for MFR. To evaluate all possible relations among the variants exceeding the significance threshold for each trait, a Venn diagram was used (Figure 4). The highest number of common variants (*i.e.*, 6,859) was between BFT and CRCL and the second highest was between BFT and MFR (*i.e.*, 2,380). To get an estimate of systemic bias, quantile-quantile plots were generated for all p-values from each GWAS (Supplementary Figure 2). As a measure of association between observed and expected p-values, lambda values were calculated for all four traits: λ_ADG_ = 1.282319, λ_BFT_ = 1.333425, λ_CRCL_ = 1.422044, and λ_MFR_ = 1.35587.

**Table 4:** Top associated genes for average daily gain (ADG), back fat thickness (BFT), meat to fat ratio (MFR), and carcass length (CRCL) identified in the GWAS. Genes incorporating or nearby the top 5 variants in the clusters are listed with chromosome and cluster numbers

Trait	SSC	Cluster no./SSC	Genes
ADG	2	1	*SHANK2*
	4	1	*RPS20*, *LYN*, *PLAG1*
	7	2	*HMGCLL1*, *TFEB*
BFT	1	1	*ZNF462*, *ENSSSCG00000005432*
	2	7	*LOC102158414*, *PGA5*, *MRPL16*, *ENSSSCG00000013151*, *ZFP91*, *CTNND1*, *ENSSSCG00000024984*, *OR9Q2*, *OR10Q1*
	4	1	*RPS20*
	7	24	*SCGN*, *LRFN2*, *DAAM2*, *C7H6orf223*, *C7H6orf132*, *RIPOR2*, *CARMIL1*, *BMP5*, *ENSSSCG00000001500*, *KIFC1*, *C6orf106*, *PPARD*, *FKBP5*, *CPNE5*, *ENSSSCG00000001574*, *TMEM217*, *LRFN2*, *MRPS10*, *TRERF1*, *RUNX2*, *RCAN2*, *MEP1A*, *ADGRF5*, *PTCHD4*, *ENSSSCG00000001734*, *PGK2*, *IREB2*, *ABHD17C*, *GSTA2*, *CRABP1*, *CRISP3*, *PRSS16*, *TBC1D2B*, *ENSSSCG00000038708*, *BCL2A1*, *E2F3*
MFR	1	3	*LOC106507123*, *TMEM245*, *SCAI*, *ABL1*, *RAPGEF1*, *CFAP77*, *DDX31*, *MAPKAP1*
	2	8	*LOC102158414*, *LOC110259166*, *LOC110259708*, *TMEM80*, *DEAF1*, *EHD1*, *MACROD1*, *ATL3*, *NAV2*, *DHCR7*, *ENSSSCG00000028537*, *CTTN*, *SHANK2*, *ENSSSCG00000036180 (KRTAP5-5-like)*, *NELL1*
	4	3	*PDE7A*, *SNTG1*, *RPS20*
	5	1	*ENSSSCG00000034097*
	7	1	*ENSSSCG00000001500*
	18	6	*PPP1R3A*, *IMMP2L*, *LRRC4*, *EXOC4*, *SND1*, *ELMO1*, *MDFIC*, *TFEC*
CRCL	1	1	*FNBP1*
	7	52	*VEGFA*, *FLRT2*, *LRFN2*, *MCTP2*, *DAAM2*, *PGF*, *SV2B*, *MAX*, *COL21A1*, *KLHL25*, *NPAS3*, *LOC110261756*, *NHLRC1*, *TPMT*, *CDKAL1*, *GMNN*, *RIPOR2*, *MDC1*, *DDX39B*, *HMGCLL1*, *ENSSSCG00000001500*, *C6orf106*, *KCTD20*, *SRSF3*, *ENSSSCG00000001612*, *FOXP4*, *TFEB*, *RCAN2*, *ADGRF1*, *MUT*, *CRISP1*, *TFAP2D*, *PKHD1*, *BNC1*, *ENSSSCG00000001827*, *TMEM266*, *NKX2-1*, *PRKD1*, *LPCAT4*, *NR2F2*, *MCTP2*, *SLCO3A1*, *ENSSSCG00000002270*, *FUT8*, *ENSSSCG00000002317*, *DPF3*, *PTGR2*, *ZNF410*, *FAM161B*, *EIF2B2*, *MLH3*, *VIPAS39*, *SPTLC2*, *ENSSSCG00000010328*, *RF01299*, *RF00100*, *HMGN4*, *NRXN3*, *ID4*, *SYNJ2BP*, *ZFP36L1*, *RAD51B*, *AVEN*, *ANG*, *GCM1*, *FOXG1*, *ENSSSCG00000033840*, *ENSSSCG00000035274*, *RSL24D1*, *NSSSCG00000036697,ENSSSCG00000037115*, *ENSSSCG00000038445*, *CEMIP*, *SLC25A21*, *SPTSSA*, *ENSSSCG00000039877*, *DIO2*, *ENSSSCG00000040930*
	17	8	*BMP2*, *JAG1*, *SPTLC3*, *TMX4*

### VEP and high impact variants

To predict functional consequences on genes the ensembl VEP tool was employed. Multiple transcripts per gene resulted in larger numbers of annotations that are reflected in the higher number of predicted effects as compared to the actual number of identified variants per trait. All inferred consequences for bonferroni corrected variants per trait and their percentage breakdown are summarized in Table 5. The large majority (over 70%) of the consequences were classified as intron variants. According to the severity of the variant consequence, intron variants are assigned to having a modifier impact, which means that predictions are difficult to be made or there is no solid evidence of impact. Variants inferred to have a disruptive impact on the protein, leading to protein truncation, loss of function or causing nonsense-mediated decay were of further interest. These significant high impact variants (Table 6) were mostly located on SSC7, with the exception of SSC2:rs1110687780 (splice donor variant) affecting *TCN1* for the trait MFR. For the BFT, the most severe consequences were located in the genes *C6orf89*, *PI16*, *DST*, and *PRIM2*, while for the CRCL disruptive impact variants were found in *NEU1*, four novel genes, *ABCD4*, *DST*, *PRIM2*, and *LPCAT4*. Notably, the same two splice donor variants affect the common genes for BFT and CRCL: *DST* and *PRIM2*. Sorting Intolerant From Tolerant (SIFT) scores were determined for all significant missense variants and are summarized in Supplementary Table 3 (Ng and Henikoff 2003).

**Table 5:** Results of variant effect prediction for the production traits average daily gain (ADG), back fat thickness (BFT), meat to fat ratio (MFR), and carcass length (CRCL). Bonferroni-corrected variants were analyzed

Predicted effect	ADG	ADG %	BFT	BFT %	MFR	MFR %	CRCL	CRCL %
Missense variant	2	0.1580	962*	0.6523*	58	0.1893	787*	0.4750*
Frameshift variant	0	0	0	0	0*	0*	6*	0.0036*
Start lost	0	0	1	0.0007	0	0	0	0
Stop gained	0	0	0	0	0	0	1	0.0006
Inframe deletion	0	0	1*	0.0007*	0	0	2	0.0012
Intron variant	936	73.9336	116815	79.2090	21556*	70.3525*	131590	79.4275
5 prime UTR variant	0	0	229*	0.1553*	89	0.2905	277*	0.1672*
3 prime UTR variant	8	0.6319	1160*	0.7866*	1242	4.0535	1543*	0.9314*
Upstream gene variant	50	3.9494	5680*	3.8514*	2195*	7.1638*	5300*	3.1991*
Downstream gene variant	44*	3.4755*	5791*	3.9267*	3442	11.2337	6893*	4.1606*
Frameshift variant, splice region variant	0	0	2	0.0014	0	0	0	0
Missense variant, splice region variant	0	0	41*	0.0278*	0	0	75*	0.0453*
Splice region variant, non coding transcript exon variant	0	0	2	0.0014	3*	0.0098*	5	0.0030
Splice region variant, 3 prime UTR variant	0	0	3*	0.0020*	3*	0.0098*	0	0
Splice region variant, intron variant, non coding transcript variant	0	0	2*	0.0014*	4	0.0131	20*	0.0121*
Splice region variant, intron variant	0	0	426*	0.2889*	41*	0.1338*	489*	0.2952*
Splice region variant, synonymous variant	0	0	21	0.0142	22*	0.0718*	28*	0.0169*
Splice donor variant	0	0	36	0.0244*	1*	0.0033	37	0.0223
Intergenic variant	109	8.6098	3318	2.2498	644	2.1018	9909	5.9811
Synonymous variant	0	0	2837	1.9237	214*	0.6984*	2751	1.6605
Intron variant, non coding transcript variant	117*	9.2417*	9636	6.5339	1060*	3.4595*	5759*	3.4761*
Non coding transcript exon variant	0	0	514*	0.3485*	66	0.2154	200*	0.1207*
Start lost, start retained variant, 5 prime UTR variant	0	0	0	0	0	0	1*	0.0006*
**Total**	**1266**		**147477**		**30640**		**165673**	

**Table 6:** Statistically significant high impact variants that were discovered in the genome wide association studies for the production traits average daily gain (ADG), back fat thickness (BFT), meat to fat ratio (MFR), and carcass length (CRCL)

Trait	High impact consequence	Variant	Position bp	Gene	Gene name
**BFT**	Start lost	SSC7:rs319855624	32544657	*C6orf89*	chromosome 7 C6orf89 homolog
	Frameshift variant, splice region variant	SSC7:._504514	32606375	*PI16*	peptidase inhibitor 16
		SSC7:._504513	32606373	*PI16*	peptidase inhibitor 16
	Splice donor variant	SSC7:rs80834233	29157904	*DST*	dystonin
		SSC7:rs327743463	28571665	*PRIM2*	DNA primase subunit 2
**MFR**	Splice donor variant	SSC2:rs1110687780	11630410	*TCN1*	transcobalamin 1
**CRCL**	Start lost, start retained variant, 5 prime UTR variant	SSC7:rs793752812	23958518	*NEU1*	neuraminidase 1
	Stop gained	SSC7:rs334442580	87783592	*novel gene*	
	Frameshift variant	SSC7:._1165873	97574140	*ABCD4*	ATP binding cassette subfamily D member 4
		SSC7:rs693811701	48561663	*novel gene*	aurora kinase A-like
		SSC7:._1068730	87783712	*novel gene*	
		SSC7:._1068731	87783718	*novel gene*	
	Splice donor variant	SSC7:rs80834233	29157904	*DST*	dystonin
		SSC7:rs327743463	28571665	*PRIM2*	DNA primase subunit 2
		SSC7:rs331245426	80150975	*LPCAT4*	lysophosphatidylcholine acyltransferase 4

### Gene set analysis

GO functional enrichment analysis revealed eleven significantly overrepresented GO terms including molecular functions (MF), biological processes (BP), and cellular components (CC). A list containing the GO terms and the associated list of genes is presented in Table 7. For BFT a GO-MF term was overrepresented and related to calcium ion binding (GO:0005509). Several olfactory receptor genes were prevalent for the GO terms assigned to MFR (*e.g.*, GO-BP GO:0007186 G-protein coupled receptor signaling pathway, GO-MF GO:0005549 odorant binding). The gene set for the CRCL trait was associated with two BP terms (GO:0001666 response to hypoxia and GO:0008283 cell proliferation) and two CC terms (GO:0045177 apical part of the cell and GO:0031410 cytoplasmic vesicle).

**Table 7:** Most significant Gene Ontology (GO) terms from DAVID for the top associated genes that were identified in genome wide association studies for the the traits back fat thickness (BFT), meat to fat ratio (MFR), and carcass length (CRCL)

Trait	Category	Term	Genes
BFT	MF	GO:0005509	*DST*, *LOC100152993*, *SCGN*, *GUCA1B*, *ITPR3*, *CIB2*, *GUCA1A*, *RASGRP2*
		calcium ion binding	
MFR	BP	GO:0007186	*OR5B3*, *LOC100623017*, *LOC106509349*, *LOC100512519*, *LOC100513457*, *OR9Q2*, *LOC100628183*, *LOC100511243*, *LOC100512154*, *LOC100514032*, *LOC100521066*, *LOC100519351*, *OR10Q1*, *LOC100511620*, *LOC106509346*
		G-protein coupled receptor signaling pathway	
	CC	GO:0016021	*ANO9*, *OR5B3*, *LOC100512519*, *LOC100519082*, *LOC100513457*, *LOC100628183*, *SIGIRR*, *LOC100512154*, *BET1L*, *LOC100521066*, *TMX2*, *OR10Q1*, *TMEM80*, *LOC100623017*, *LOC106509349*, *OR9Q2*, *LOC100511243*, *ZDHHC5*, *ATL3*, *LOC100514032*, *LRRC4*, *PPP1R3A*, *LOC100519351*, *LRRN3*, *LOC100511620*, *STX3*, *LOC100521938*, *CCDC136*, *LOC106509346*, *NRXN2*
		integral component of membrane	
	CC	GO:0005886	*OR5B3*, *EHD1*, *LOC100623017*, *LOC106509349*, *LOC100512519*, *OR9Q2*, *LOC100513457*, *LOC100628183*, *CTNND1*, *LOC100511243*, *ELMO1*, *LOC100512154*, *ZDHHC5*, *LOC100514032*, *LOC100521066*, *LOC100519351*, *STX3*, *LOC100511620*, *RABEPK*, *LOC106509346*, *RASGRP2*
		plasma membrane	
	MF	GO:0004930	*OR5B3*, *LOC100623017*, *LOC106509349*, *LOC100512519*, *LOC100513457*, *OR9Q2*, *LOC100628183*, *LOC100511243*, *LOC100512154*, *LOC100514032*, *LOC100521066*, *LOC100519351*, *OR10Q1*, *GPR141*, *LOC100511620*, *LOC106509346*
		G-protein coupled receptor activity	
	MF	GO:0004984	*OR5B3*, *LOC100623017*, *LOC106509349*, *LOC100512519*, *LOC100513457*, *OR9Q2*, *LOC100628183*, *LOC100511243*, *LOC100512154*, *LOC100514032*, *LOC100521066*, *LOC100519351*, *OR10Q1*, *LOC100511620*, *LOC106509346*
		olfactory receptor activity	
	MF	GO:0005549	*OR5B3*, *LOC100623017*, *LOC106509349*, *LOC100513457*, *OR9Q2*, *LOC100628183*, *LOC100512154*, *LOC100514032*, *LOC100521066*, *LOC100519351*, *OR10Q1*, *LOC100511620*, *LOC106509346*
		odorant binding	
CRCL	BP	GO:0001666	*ANG*, *TGFB3*, *PGF*, *PLAT*, *VEGFA*
		response to hypoxia	
	BP	GO:0008283	*FURIN*, *FAM83B*, *ZFP36L1*, *MORF4L1*, *BYSL*, *RASGRF1*
		cell proliferation	
	CC	GO:0045177	*ADGRF5*, *VASH1*, *PLAT*, *HOMER2*, *BYSL*
		apical part of cell	
	CC	GO:0031410	*ANG*, *ADGRF5*, *FES*, *NEU1*, *GRM4*, *RHGC*
		cytoplasmic vesicle	

## Discussion

### Genotyping strategy

**Figure 5:**
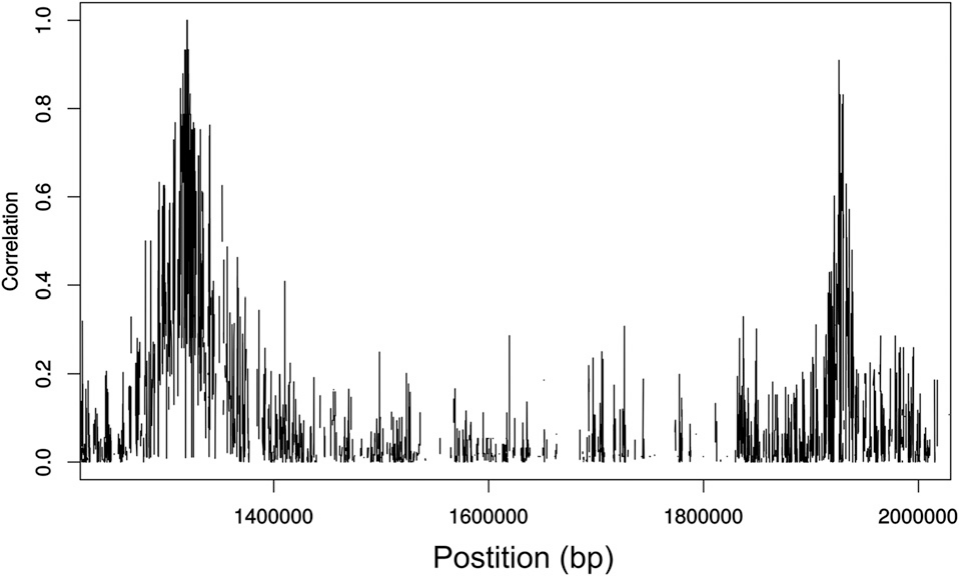
Imputation accuracy on SSC2 between positions 1,250,000 and 2,000,000. *IGF2* is located between bp 1,469,183 and 1,496,417.

The genotyping strategy that we developed for this study is outlined in Figure 1. Briefly: 24 F0 pigs were subjected to high coverage Illumina short read sequencing and in addition 91 F1 animals were sequenced at low coverage and imputed to high coverage WGS levels in order to allow phasing. 2657 F2 animals were chip genotyped and imputed using a merged dataset of F0 and imputed F1 as reference-panel. All imputation steps involved pedigree information. Opposed to a population-based strategy this approach does not rely on a large reference-panel but on the relatedness of individuals. In general, the genotyping strategy can be considered reliable since the majority of the QTL identified were already described for the four traits analyzed in this study (cross-reference with Pig QTL database (Hu *et al.* 2019)). Nevertheless, we expected to identify a variant that was associated with muscle mass and fat deposition in exon 2 of *IGF2*, which has been extensively described to influence muscle development (Nezer *et al.* 1999). The absence of *IGF2* associated variants can be explained by a local drop in coefficients of determination from an average of R^2^ = 0.22 to R^2^ = 0.03 in the genomic region where *IGF2* resides (SSC2 1469183 – 1496417 bp, Figure 5). It must be pointed out that those coefficients of determination cannot be used to draw conclusions about the actual accuracy of the imputation. Since no pedigree information was included in the simulation, it can solely be used to identify local inaccuracies, which were most likely due to assembly errors in the reference genome.

The genotyping approach presented in this study can be considered a reasonable strategy to radically increase the marker density of large F2 populations to WGS levels. By sequencing the founder individuals with high coverage and the F1 with low coverage, which are only a fracture of the number of F2 animals, the approach provides an affordable opportunity to improve the power and potential of otherwise obsolete datasets. Due to the relatedness of the animals deep sequencing of only a few animals is necessary, rendering it economically attractive.

### Cluster identification and exploratory analysis

To fully exploit the potential of the four resource populations, the crosses were pooled and further used for conducting GWAS. The increased sample size together with the increased marker density ensures a high resolution that might allow the pinpointing of more specific causative genes and mutations. Further experiments, *e.g.*, Sanger sequencing of promising regions could elaborate on that. Designing F2 populations implies that the LD-blocks are longer, a fact that is counteracted to some extent by jointly analyzing the four designs. Lambda values of 1.282319 to 1.422044 point to a moderate degree of p-value inflation in the GWAS, which is most likely caused by the usage of WGS data and a LOCO GWAS approach. However, to exploit the whole depth and power of the dataset we chose a LOCO analysis approach. To further comprehend the closely linked association signals from GWAS, the following approach was employed: i) clusters incorporating strong evidence for trait-associated chromosomal regions were defined, ii) the effect of the significant variants was predicted, and iii) a gene set analysis was employed to identify sets of genes jointly associated with the traits of interest.

The quantitative traits considered for this study have been investigated in the past and are mostly well represented in the Pig QTL database (Hu *et al.* 2019), except for MFR. The clusters assigned to each trait were compared with the QTL regions from the database. For MFR, additional fat-related traits (*e.g.*, fat percentage in the carcass and fat-cuts percentage) were considered in order to allow an adequate comparison given that the trait has few records in the database and the trait definition can be country dependent. Most of the clusters overlapped or were in the vicinity of the previously reported QTL. This was expected as the database has been recently updated and also includes our previous results (Blaj *et al.* 2018) using SNP chip data and three out of the four pig populations which were taken into account here. Some of the earlier reported QTL in the database spread over large genomic regions (*e.g.*, > 5Mb). It is assumed that many of these large QTL regions might in fact not be due to a single mutation, thus representing haplotype effects caused by several causative variants (Andersson 2009). In the current study, we were able to assign numerous clusters within these regions, which implies that a higher genomic resolution was achieved and that it may be possible to disentangle distinct quantitative trait nucleotides.

Conditional association analyses by including the top variant as a fixed effect in the MLMA were carried out in order to gather statistical evidence for putative causality (Cohen-Zinder *et al.* 2005) and was specifically applied to CRCL and BFT on SSC7. This chromosome exhibits the highest number of clusters (SM with Clusters) and the highest association signals. By including the top variant (rs81228492) for BFT, only one well-supported peak was above the significance threshold (Supplementary Figure 1) meaning that there is additional genetic variation within this region. Similarly, for CRCL the two top variants (rs333021601 and rs319044994) representing the two different significant genomic regions were included alternatively in the model. After fixing the effect of the latter variant, the surrounding significant region disappeared, pointing to the possibility that there could be only one QTL responsible for CRCL on SSC7 around the 99 Mb region. An alternative or additional explanation could be the presence of long LD blocks, long-range LD and/or various epistatic interactions among the loci. The overlap among the BFT and CRCL significant variants (see Figure 3 and Figure 4) localized mostly in the genomic region 24-32 Mb indicate the existence of pleiotropic loci for the two traits. When conditioned on the top BFT variant (rs81228492) as a fixed effect for a MLMA on CRCL and the top CRCL variant (rs333021601) for MLMA on BFT, the initially associated clusters and those nearby dropped in the intensity of the association signals (Supplementary Figure 1), supporting the presence of pleiotropic loci. It is also noteworthy that CRCL might be influenced by the number of thoracolumbar vertebrae (Rohrer *et al.* 2015). Since the variant that has been associated with a higher number of vertebrae is a large Indel in intron 1 of the *VRTN* gene (Fan *et al.* 2013) we were not able to discover this variant since the genotyping pipeline applied in this study does only cover small INDELs.

In order to gain insight into the possible genetic mechanisms that control the traits, an enrichment analysis of the gene function was performed with DAVID, prioritizing on the GO terms. The GO-MF calcium ion binding term found for BFT supports the relationship between the calcium ion, food intake and lipid metabolism previously described in the literature (Cui *et al.* 2017). Furthermore, one of the genes in this group is *DST*, a strong candidate gene for which high impact variants were found via VEP, which is discussed in detail below. A GO-BP term related to cell proliferation comprised the *FAM83B* gene, which is the gene incorporating the top variant found for CRCL. Interestingly, the majority of the genes included in the over-represented terms for MFR were olfactory receptors. This enrichment is a consequence of the MFR-identified clusters overlapping regions that are rich in various olfactory receptor genes. This particular gene family is known to have significant expansion throughout time within the *Sus Scrofa* genome (Nguyen *et al.* 2012).

### Variant effect prediction

#### ADG:

A QTL for ADG found on SSC7 comprises 115 statistically significant intron variants and 83 variants upstream (min. p-value 8.71 × 10^−14^) of the *HMGCLL1* gene, which was shown by Comuzzie *et al.* to be associated with childhood obesity in the Hispanic population and to influence creatinine levels. Another QTL on SSC2 contains 112 intron variants in *SHANK2* (min. p-value 1.33 × 10^−12^). *SHANK2* was also shown to be associated with childhood obesity in the same study and to have an influence on estradiol blood concentrations (Comuzzie *et al.* 2012). A third QTL on SSC4 harbors 2 intron and 12 downstream variants (min p-value 1.06 × 10^−13^) affecting *LYN*, which encodes for the LYN proto-oncogene, which was also identified by Comuzzie *et al.* and correlated with the amount of fat mass in obese children (Comuzzie *et al.* 2012). Six additional variants in the QTL on SSC4 (min. p-value 2.44 × 10^−12^) lie in an intergenic region 13,463 – 14,460 bp downstream of *RPS20*, a gene which in interplay with *GNL1* is critical for cell growth (Krishnan *et al.* 2018). Another likely candidate SNP to influence ADG is an intron variant in the *PLAG1* transcription factor (p-value 1.32 × 10^−11^), which is a regulator of *IGF2* expression (Zatkova *et al.* 2004).

#### BFT:

A QTL for BFT with a very prominent peak was detected on SSC7. The SNP with the lowest p-value (6.63 × 10^−54^) is an intron variant in gene *C6orf106*. *C6orf106* is a target of the human miRNA has-miR-192, which has been identified to have regulatory functions in type 2 diabetes mellitus (Cui *et al.* 2016). The second top scoring SNP is an intron variant in the *RIPOR2* gene (p-value 4.34 × 10^−50^). *RIPOR2* expression and protein levels are upregulated during muscle cell differentiation in human fetal muscle cells (Yoon *et al.* 2007). Another gene containing top scoring variants on SSC7 is *KIFC1* (7 intron variants, min p-value 3.12 × 10^−47^). Overexpression of *KIFC1* promotes cell proliferation in non-small cell lung cancer (Liu *et al.* 2016). 21 intron and 8 downstream variants in *BMP5* (min. p-value 1.91 × 10^−29^), which induces cartilage and bone formation (Wozney *et al.* 1988), are also located in a cluster on SSC7. 6 variants downstream of the aforementioned RPS20 (min. p-value 1.90 × 10^−15^) were found in the cluster on SSC4.

#### MFR:

GWAS for the MFR trait revealed a strong QTL on SSC2 with variant rs81327136 upstream of *KRTAP5-5-like* being the most significant (p-value 1.59 × 10^−23^). Of 72 variants 6 were located in *KRTAP5-5-like* introns and 66 in the vicinity of the gene. *KRTAP5-5* was shown to control cytoskeletal function and cancer cell vascular invasion (Berens *et al*. 2017). Other variants found in clusters on SSC2 are located in or adjacenct to *DEAF1* (8 intron variants, min p-value 3.47 × 10^−29^), which is a transcription factor that regulates proliferation of epithelial cells (Barker *et al.* 2008) and that forms a dominant-negative splice isoform in type 1 diabetes, which correlates with disease severity (Yip *et al.* 2015). Clusters on SSC2 also harbor variants associated with *SHANK2* (1,714 intron variants, 3 5′ UTR variants, min p-value 2.18 × 10^−26^) and *CTTN* (188 up- and downstream variants, min p-value 1.53 × 10^−25^). *CTTN*’s protein product Cortactin binds to and is indirectly phosphorylated by obesity factor PTP1B (Stuible *et al.* 2008). A noteworthy intron variant is located in the vitamin D pathway gene *DHCR7* (p-value 3.06 × 10^−25^), which has been associated with obesity traits in humans (Vimaleswaran *et al.* 2013). A total of 14 *DHCR7* intron variants were above the significance threshold. A less prominent QTL on SSC4 harbors variants in or close to the aforementioned genes *RPS20* (17 downstream variants, min p-value 1.51 × 10^−25^) and in *SNTG1* (19 intron variants, min p-value 1.48 × 10^−14^), which has been associated with type 2 diabetes (Ban *et al.* 2010). A third, rather minor QTL on SSC18, contains 21 variants downstream of *MDFIC* (min p-value 1.97 × 10^−15^), a gene which has been linked to improved piglet birth weight (Zhang *et al.* 2014). 25 intron and 42 downstream variants were found for the *PPP1R3A* gene (min p-value 6.92 × 10^−15^), which in a whole exome sequencing study was found to be associated with type 2 diabetes in a Mayan population (Sánchez-Pozos *et al.* 2018).

#### CRCL:

In the GWAS for CRCL 52 clusters were identified on SSC7. Although not located in one of the clusters, the two lowest p-values (min p-value 5.40 × 10^−49^) were found in the intron and coding region (silent mutation) of *FAM83B* (or *C6orf143*) respectively. A total of 62 significant variants in FAM83B were discovered comprising of 60 intron variants, 1 silent mutation, and 1 missense mutation. Cipriano *et al.* demonstrated that overexpression or mutation of FAM83B leads to EGFR hyperactivation by direct interaction and consequent hyperactivation of the EGFR downstream effector phospholipase D1, which was previously associated with BMI in humans (Davenport *et al.* 2015). An intron variant in the *RIPOR2* gene with a p-value of 5.08 × 10^−47^ is the same SNP, which was found in the GWAS for BFT. A total of 85 mostly intronic *RIPOR2* variants were found for the CRCL trait. A second, less prominent QTL on SSC7 harbors 9 intron, 12 downstream and 317 upstream variants (min p-value 3.62 × 10^−31^), which have been assigned to the *RSL24D1* gene. *RSL24D1* has been identified as a potential target in familial hypercholesterolemia (Li *et al.* 2015). One of the clusters identified for CRCL on SSC17 contains 230 variants 122,416-126,520 bp downstream of BMP2 (min p-value 7.21 × 10^−38^), a bone formation inducing factor (Wang *et al.* 2013). In addition, 18 intron and 114 variants upstream of *TMX4* were discovered. *TMX4* was associated with feed conversion ratios in chickens (Shah *et al.* 2016).

#### High impact variants:

Various high impact variants were discovered by variant effect prediction. A splice donor variant (rs80834233) in *DST*, the gene encoding Dystonin, is associated with BFT (p-value 1.98 × 10^−19^) and CRCL (p-value 1.25 × 10^−19^). Knockout of DST leads to intrinsic muscle weakness and instability of skeletal muscle cytoarchitecture in mice (Dalpé *et al.* 1999). Variant rs793752812 leads to a probable start codon loss in *NEU1* and is associated with CRCL (p-value 1.49 × 10^−12^). A deficiency of the *NEU1* gene product Neuraminidase 1 leads to vertebral deformities in humans (Sphranger *et al.* 1977), which is reasonable considering CRCL is largely determined by the number of vertebrae. Furthermore one frameshift variant in *AURKA* (rs693811701, p-value 2.95 × 10^−12^) and one splice donor variant in *NUTM1* (rs331245426, p-value 1.38 × 10^−9^), both oncogenes (Umene *et al.* 2015) (Schaefer *et al.* 2018), are associated with CRCL. The splice donor variant rs1110687780, which affects the gene coding for placenta-specific protein 1-like, was detected in the GWAS for MFR. In humans, *PLAC1* has been found to be highly expressed in various types of tumors (Koslowski *et al.* 2007).

### Application of results in breeding programs and follow up studies

Functional validation studies based on appointed candidate genes and genetic variants will be considered in follow-up studies. Besides understanding the underlying molecular mechanisms of ADG, BFT, MFR and CRCL, the results of GWAS can render a substantial increase in the reliability of genomic predictions in breeding programs. This concept was demonstrated in several studies in cattle (Brøndum *et al.* 2015; Porto-Neto *et al.* 2015; van den Berg *et al.* 2016) and in *Drosophila melanogaster* (Ober *et al.* 2015) by including pre-selected variants from GWAS results in the prediction models. Even though implementing genomic selection is becoming a common practice, the usage of marker-assisted selection or genomic screening is not obsolete pointing out that the identification of relevant genetic markers via GWAS and post-GWAS analyses is still of practical importance in pig breeding.

### Conclusion

Putting the results of previous simulation studies to test, we conducted GWAS in four pooled F2 designs, which have been imputed to sequence level based on high coverage founder and low coverage F1 sequencing. We found that by pooling the designs the sequence level marker density can be exploited efficiently. QTL for four well-characterized traits were identified in agreement with previous mapping studies and candidate genes and pathways were unraveled, that should be subject to further studies. Thus, the approach applied herein is a feasible strategy to efficiently utilize extremely well phenotyped experimental designs that have been established in the past.
